# Remote Assessment of Depression Using Digital Biomarkers From Cognitive Tasks

**DOI:** 10.3389/fpsyg.2021.767507

**Published:** 2021-12-15

**Authors:** Regan L. Mandryk, Max V. Birk, Sarah Vedress, Katelyn Wiley, Elizabeth Reid, Phaedra Berger, Julian Frommel

**Affiliations:** ^1^Interaction Lab, Department of Computer Science, University of Saskatchewan, Saskatoon, SK, Canada; ^2^Systemic Change Group, Department of Industrial Design, Eindhoven University of Technology, Eindhoven, Netherlands

**Keywords:** depression, digital biomarkers, digital phenotyping, assessment, mental health

## Abstract

We describe the design and evaluation of a sub-clinical digital assessment tool that integrates digital biomarkers of depression. Based on three standard cognitive tasks (D2 Test of Attention, Delayed Matching to Sample Task, Spatial Working Memory Task) on which people with depression have been known to perform differently than a control group, we iteratively designed a digital assessment tool that could be deployed outside of laboratory contexts, in uncontrolled home environments on computer systems with widely varying system characteristics (e.g., displays resolution, input devices). We conducted two online studies, in which participants used the assessment tool in their own homes, and completed subjective questionnaires including the Patient Health Questionnaire (PHQ-9)—a standard self-report tool for assessing depression in clinical contexts. In a first study (*n* = 269), we demonstrate that each task can be used in isolation to significantly predict PHQ-9 scores. In a second study (*n* = 90), we replicate these results and further demonstrate that when used in combination, behavioral metrics from the three tasks significantly predicted PHQ-9 scores, even when taking into account demographic factors known to influence depression such as age and gender. A multiple regression model explained 34.4% of variance in PHQ-9 scores with behavioral metrics from each task providing unique and significant contributions to the prediction.

## 1. Introduction

Depression is currently the leading cause of disability around the world (Friedrich, 2017) and contributes heavily to the estimated US $2.5–8.5 trillion in lost output globally from mental, neurological, and substance use disorders (Wykes et al., 2015). Diagnosing depression involves clinicians who employ interview techniques, questionnaires, and test batteries that follow standardized manuals, such as the DSM-V (American Psychiatric Association, 2013). As a complement to these techniques, digital biomarkers of depression—that is, measurable responses gathered from digital devices and used to reliably predict the incidence of depression—could help inform clinician assessment, particularly when they can be gathered easily, unobtrusively, and outside of the clinical context. Digital biomarkers of depression could enhance clinical treatment (Mohr et al., 2017), including through timely identification for early intervention, ongoing assessment during treatment, and by reducing disparities in access to assessment due to factors such as geography or income (Kumar and Phookun, 2016; Naslund et al., 2017). Digital biomarkers additionally support assessment for subclinical populations—that is, people who live with symptoms of depression that may not meet criteria for a DSM-V diagnosis, but who are prevented from achieving their potential, leading their fullest lives, and for whom symptoms may escalate in severity if left untreated. Further, digital biomarkers of depression deployed at a large scale could be used for population screening or prevalence estimations that are not currently possible with traditional clinician-intensive approaches (Gillan and Daw, 2016).

Previous approaches in the design of digital biomarkers for assessing depression have harnessed data from a variety of digital sources, including from smartphones and social media use. For example, Saeb et al. (2015) showed that location features drawn from 2 weeks of mobile phone use (e.g., location variance, location entropy, and regularity over 24-h) along with phone usage metrics (e.g., duration, frequency of use) were related to depressive symptoms. The authors argued that predicting depression through passively sensing daily behaviors is feasible in principle, as daily routines include behaviors that mark presence of depression (e.g., social behaviors or sleep behaviors), which can be sensed by smartphones. Using various features extracted from mobile phones (e.g., location, physical activity, phone calls, text messages, WiFi), researchers have trained machine learning models to predict aspects of self-reported depression or depressive symptoms (Canzian and Musolesi, 2015; Farhan et al., 2016; Wahle et al., 2016; Wang et al., 2018). For example, using a variety of smartphone sensors (e.g., bluetooth, screen status, call logs, location sensing) over the course of a college semester, Xu et al. (2019) were able to predict whether students were likely to report high scores on Beck's Depression Inventory (BDI-II: Beck et al., 1996) at the end of the semester. Further, Chikersal et al. (2021) showed that this prediction could be accurately made 11–15 weeks before the end of the semester, allowing time for preventative interventions. Passively sensing explicit behaviors through smartphone use has been shown as a promising approach for augmenting the detection of depression.

In addition to passively detecting behaviors, researchers have investigated passively detecting communications for features that mark depression. In particular, social media posts contain content that has been used to predict the presence of major depression (De Choudhury et al., 2013) from sources such as Twitter (e.g., Tsugawa et al., 2015), Reddit (e.g., Aladağ et al., 2018), Facebook (e.g., Park et al., 2013), Sina Weibo (e.g., Cheng et al., 2017), and Instagram (e.g., Reece and Danforth, 2017). And as with smartphone sensing approaches, social media posts are also used to predict the presence of symptoms associated with depression, such as suicidal ideation (e.g., Burnap et al., 2015; Shing et al., 2018), and the severity of the mental illness (e.g., Chancellor et al., 2016). Although semantic analysis of the posts themselves are often used as a feature in sensing depression, other metrics derived from behavior (e.g., activity, followers, networks), posted images, or sentiment analysis have also contributed to machine learning models using social media data (De Choudhury et al., 2013).

What these methods have in common is that they use computational approaches to identify ways in which people with depression communicate or behave differently than those without depression (Mandryk and Birk, 2019). Researchers generally employ a “bottom-up” machine learning (LeCun et al., 2015) approach that is naive to known effects of depression on cognition or behavior, but instead harnesses activity traces left behind by natural interactions with the world to build blackbox models that classify people, using ground truth labels of depression, such a clinical diagnosis or self-report scales. However, when behavioral or cognitive correlates of depression are already known, a contrasting approach (Mandryk and Birk, 2019) is to develop custom software tools that monitor people's responses (e.g., reaction time, performance, decisions), and then use statistical approaches to predict the likelihood of depression. For example, this custom tool approach has been successfully used to assess dementia on a large scale https://glitchers.com/project/sea-hero-quest/.

In the domain of depression, there has been significant research investigating behavioral and cognitive differences of people with a diagnosis of depression, with remitted depression, or with medicated treatment of depression, as compared to healthy control groups. For example, studies demonstrate that people with depression exhibit reduced visual contrast acuity or sensitivity (Bubl et al., 2009, 2010; Fam et al., 2013). Studies have suggested that people with depression have a recall bias that preferences negative autobiographical recall (Brittlebank et al., 1993) and an attention maintenance bias toward dysphoric images and sad faces (Suslow et al., 2020). Further, a significant body of work has focused on cognitive differences between people with depression and healthy controls and has found deficits, especially on measures of attention, executive function, memory, and psychomotor speed (Tavares et al., 2003; Chamberlain and Sahakian, 2006). Additionally, some of these attentional deficits have been shown to persist, even when patients have recovered fully, according to clinical diagnosis (Silverstein et al., 1994; Chamberlain and Sahakian, 2006). A diagnostic criterion for major depressive disorder is a “diminished ability to think or concentrate” (American Psychiatric Association, 2013), which can include difficulties with all types of attention. Depression has been linked to impairments in selective attention (the ability to attend to relevant information and ignore irrelevant stimuli), sustained attention (the ability to focus on something for a continuous amount of time) and divided attention (the ability to attend to multiple things at once) (American Psychiatric Association, 2013). Studies have also shown that people with depression demonstrate attentional biases toward negative information (MacLeod et al., 1986; Peckham et al., 2010).

Traditionally, measuring attention has been done using cognitive tasks in which participants are shown stimuli and asked to respond in different ways, while their reaction times and accuracy are measured. A variety of cognitive tasks rely on attention, such as the Stroop task (selective attention) (Kertzman et al., 2010; Keller et al., 2019), Continuous Performance Task (sustained attention) (Shalev et al., 2011; Conners, 2014), and bimodal tasks (divided attention) (Thomas et al., 1998). When comparing participants with depression to healthy controls on these cognitive tasks, those with depression generally demonstrate slower response times. Some of these differences may be due to psychomotor slowness or low mood rather than impairments specific to attention (Kertzman et al., 2010; Keller et al., 2019), though further research correlates depression with impairments specific to attentional control and executive functions (Snyder, 2013; Rock et al., 2014). These studies have found impairments correlated with updating (the ability to take new information into working memory), shifting (the ability to allocate attention to whatever is most relevant at the time), and inhibition (the ability to prevent irrelevant stimuli from impairing performance) (Snyder, 2013). For example, meta-analyses have found that depressed participants show significant deficits compared to healthy controls on the D2 Test of Attention, Delayed Matching to Sample Task, and Spatial Working Memory Task (Rock et al., 2014; Wang et al., 2020).

Although previous research has shown a variety of differences in measures of attention between people with depression and healthy controls, using these tasks for assessment can be complex. In particular, cognitive tasks that have traditionally produced robust experimental effects may not reliably correlate with individual differences, an effect that Craig, Hedge, and Sumner call ‘the reliability paradox’ (Hedge et al., 2018b). For this reason, some tasks traditionally associated with depression may not be suitable for assessment, such as emotional Stroop tasks (Eide et al., 2002) or other tasks based on attentional biases (MacLeod et al., 2019; Gladwin et al., 2020).

There are also challenges with gathering data related to attention *in situ* for remote assessment. Gathering data *in situ*—rather than in controlled laboratory contexts—presents challenges to researchers. For example, differences in hardware (e.g., screen size, display resolution, visual angle, refresh rate) make conducting research that relies on visual stimuli less controlled than experimenters are accustomed to. Differences in software settings (e.g., control-display gain, cursor acceleration) make conducting research on psychomotor tasks less controlled than in a laboratory. Although progress has been made in the last decade in research methods that support online experiments (Buhrmester et al., 2011, 2018; Mason and Suri, 2012), particularly for challenging psychomotor tasks, e.g., Peirce, 2007, the lack of control *in situ* still raises challenges for gathering data related to human attention. The lack of control over the auditory environment (e.g., sirens, construction outside, television or music playing), the interruptions of family members or pets, the presence of children, and the propensity to multitask—both on and off the computer—all make the assessment of attention *in situ* a challenging task. However, there are consistent and persistent associations of depression with error measures from tests of attention that we propose may be more robust to *in situ* assessment than measures related to reaction time, response latency, or speed of performance, as timing measures may be susceptible to variations in computing systems, like display latency or input lag.

In this paper, we harness depression-related differences in errors within attention tasks to design and evaluate a sub-clinical digital assessment tool that integrates digital biomarkers of depression. Based on three standard cognitive tasks (D2 Test of Attention: Brickenkamp, 1962; (Brickenkamp and Zillmer, 1998), Delayed Matching to Sample Task: Ferster, 1960; Sahakian et al., 1988; Robbins et al., 1997; Jäkälä et al., 1999, Spatial Working Memory Task: Owen et al., 1990; De Luca et al., 2003) on which people with depression have been known to perform differently than a control group (Rock et al., 2014; Wang et al., 2020), we designed a digital assessment tool that can be deployed outside of laboratory contexts, in uncontrolled home environments on computer systems with widely varying system characteristics (e.g., display resolution, input devices). We evaluated the assessment tool in two online studies—with participants in their own homes completing the task on their own digital devices—to show that the assessment tool can significantly predict scores from the Patient Health Questionnaire (PHQ-9: Kroenke et al., 2001)—a standard self-report tool for assessing depression in clinical contexts.

## 2. Materials and Methods

### 2.1. The Digital Assessment Tool

Our digital tool embeds three standard tests of attention in a single assessment.

**Figure 1:**
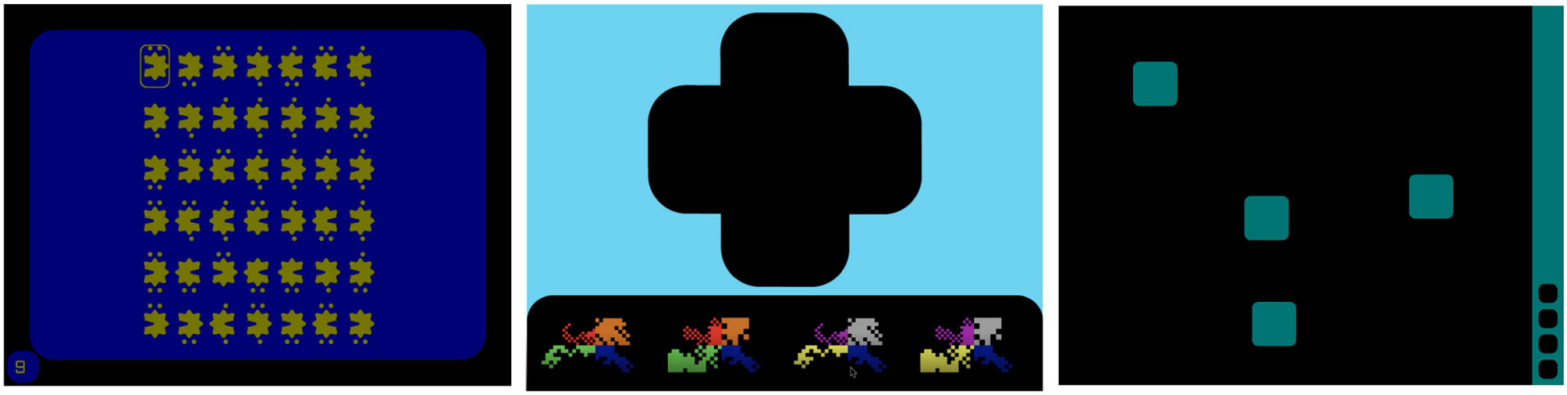
Screenshots of the three attention tasks used in the studies: The D2 test of attention **(left)**, delayed matching to sample **(middle)**, and spatial working memory **(right)**.

The *D2 Test of Attention* Brickenkamp, 1962; (Brickenkamp and Zillmer, 1998) measures sustained and selective attention. It uses rows of hard-to-distinguish stimuli—historically consisting of the letters d and p with 1–4 markings at the top or bottom. People are instructed to mark each item that fits a certain description. The test has been developed and is mostly used in pen-and-paper form. Our implementation used shapes with notches on the left or right and 1 to 4 dots in a 7 by 6 grid (see Figure 1, left). The correct stimulus was defined as those with 2 dots and a notch on the left side. Participants navigated between the stimuli using the left and right arrow keys on their keyboards and had to select those that correspond to the correct form using the “Z” key. Participants were given 15 s to complete a single page of the D2 task, after which they were given a break before progressing to the next round; there were 20 rounds of the D2 task in total. Our distribution of targets followed (Brickenkamp and Zillmer, 1998): on each page, there were correct targets and distractors that were either the correct notch and incorrect dots, incorrect notch and correct dots, or incorrect notch and dots.

The *Delayed Matching to Sample* (DMTS) test (Ferster, 1960; Sahakian et al., 1988; Robbins et al., 1997; Jäkälä et al., 1999) measures visual matching ability and short-term working memory. Participants are shown a visual object as a prompt and instructed to remember it as they would be required to identify it later. After a short delay, four choice patterns appeared, with one of them exactly matching the prompt and the other three being distractors. Similar to Sahakian et al. (1988), the visual object consisted of 4-quadrant abstract patterns that used one color and one form per quadrant (see Figure 1, middle). One of the four choice patterns was identical to the prompt. One of the three distractors was a novel distractor, differing in both color and form from the prompt. The remaining two distrators were ‘partial distractors’; one had the colors of the prompt but the form of the novel distractor, while the other had the form of the prompt but the colors of the novel distractor. As with Robbins et al. (1997), each of the four choice patterns had one random quadrant in common (both color and form) to discourage mnemonic strategies based on remembering the color and shape of a single quadrant. In our implementation, the prompt was shown for 4,500 ms and we used four different delays (0, 1, 4, and 12 s) between the prompt and the four choices. During the delay, a mask was presented, which was an animated rotation through distractor images. Participants selected the correct choice using mouse clicks and were given feedback in the form of red crosses and green checkmarks. If an incorrect choice was made, participants were required to continue selecting choices until the correct (prompt) stimulus had been chosen. For each delay, participants were given 10 prompts (40 in total).

The *Spatial Working Memory* (SWM) task (Owen et al., 1990; De Luca et al., 2003) assesses a participant's ability to retain and manipulate visuospatial information. The task begins with a set of boxes on display; participants have to search through the boxes to locate a hidden token. This repeats over several “sequences” (equal to the number of boxes); as tokens are discovered, they fill up a column on the right hand side of the screen (see Figure 1, right). Each box houses only one token per set of sequences, and participants are instructed that once a token has been found in a particular box, that box would not be used again to hide a token. After the set of sequences has been completed, the display is cleared and the position of the boxes are changed in the next trial to discourage the use of stereotyped search strategies. In our implementation, participants opened boxes using mouse clicks to search for the token until finding the correct box. After opening a box, an animation revealed whether the box was empty or contained the token. We used 6 different difficulty levels corresponding to different numbers of boxes to choose from in a trial (4, 6, 8, 10, 12, 14). Participants completed one trial at each difficulty level.

In our assessment tool, participants were randomly assigned a shape—a form and color combination that was used throughout the system. This shape was used in the D2 task with the notches on either side and dots above and below, in the DMTS as the background, and in the SWM as the token to be searched (see Figure 1). We chose this abstract shape approach intentionally as even a small change made to an established stimuli can affect performance in computerized assessments of attention (Price et al., 2015). Prior to each task, a step-by-step tutorial was provided to instruct participants on the goal of, and interaction within, the task. Following the tutorial, the task was completed.

### 2.2. Measures

We collected indicators derived from the participants' interaction with our digital assessment tool and self-report measures.

#### 2.2.1. Digital Assessment Tool Measures

##### 2.2.1.1. D2 Test of Attention

From the D2 test of attention, we calculated performance measures (summed across the 20 repeated trials) including: the *number of items* processed in the time limit, the *number of correctly marked stimuli, number of omission errors* (false negatives), *number of commission errors* (false positives), *total number of errors* (sum of omission and commission errors), and the *error rate* (number of errors per time).

##### 2.2.1.2. Delayed Matching to Sample

We calculated the number of correct choices and latency (i.e., response time) for the four different levels of delay, across the 10 repeated trials. We then calculated our measures across the four delay levels (sum), including: *number of correct choices, average latency, number of color errors* (when participants selected an object with correct form but incorrect color), *number of shape errors* (when participants selected an object with incorrect form but correct color), *number of color+shape errors* (when participants selected an object with incorrect color and incorrect form).

##### 2.2.1.3. Spatial Working Memory

The SWM task provides three types of outcome measures. Searching any box more than once within a sequence results in a within search error. Between search errors occur when returning to search an already emptied box in a trial. We calculated the sum of both within and between errors at each of the 6 difficulty levels individually. From this, we calculate the measures: *number of between errors* and *number of within errors*. In addition to errors, the SWM task allows calculation of a *strategy score*, (lower=better), which refers to the search strategy that is used to initiate searching. It is calculated as the sum of the different starting boxes. We calculated one total strategy score across all levels.

#### 2.2.2. Self-Report Measures

We collected several self-report measures including the participants' demographics, whether they had vision impairments, corrected vision, color blindness, or motor impairments (potentially affecting the ability to control our digital toolbox), whether they had been diagnosed with depression, anxiety, or bipolar disorder, whether they took medications for these conditions, and optional descriptions for diagnosed conditions and medications.

*PHQ-9*: We assessed self-reported depression using the Patient Health Questionnaire (PHQ-9: Kroenke et al., 2001)—a standard self-report tool for assessing depression in clinical contexts. It is the 9-item depression module of the Patient Health Questionnaire and can be self-administered (Spitzer et al., 1999; Kroenke et al., 2001). Participants rated the frequency (“Over the last 2 weeks, how often have you been bothered by any of the following problems?”) of 9 symptoms (e.g., “Feeling down, depressed or hopeless.”) on 4-point scales (0 = “Not at all,” 1 = “Several days,” 2 = “More than half of the days,” 3 = “Nearly every day”). The total score (sum of all scores) can range from 0 to 27, is a severity measure for depression (Kroenke et al., 2001), and represents the depression indicator that we predict with our assessment tool and refer to as *PHQ-9 score* for brevity. As the PHQ-9 was developed as a screening tool, the score is converted into a *level* that is used to determine the severity of the symptoms. As we also aim for biomarkers to be used as a screening tool, we predict the PHQ-9 score itself, which can be converted to the level later. The PHQ-9 includes an additional item about the difficulty resulting from the symptoms, which participants answered but was not used in the analysis.

### 2.3. Participants and Procedure

We deployed the experiment using an open-source software framework (Johanson, 2020), hosted on a University-owned data server. Participants were recruited from Amazon's Mechanical Turk (MTurk), which is an online marketplace that allows researchers to deploy studies through Human Intelligence Tasks (HITs) to diverse populations (Buhrmester et al., 2011). MTurk has been shown to be useful in behavioral research for its wide range of uses, diverse participant pool, speed, cost, and accessibility (Buhrmester et al., 2018), with valid data when precautions are taken (Mason and Suri, 2012). Upon accessing the HIT, participants provided informed consent, answered the demographic questionnaires and the trait inventories, completed a color blindness test, completed the digital assessment tool, and then completed scales evaluating the experience of using the tool. Finally, they were debriefed as to the purpose of the study, and given the option to withdraw their data (no participants chose to withdraw). Ethical approval for the studies was obtained from the Behavioural Ethics Research Board at the University of Saskatchewan.

In Study One, participants were randomly assigned to complete one of the three tasks. In Study Two, participants completed all three tasks. Because our goal was not to compare the tasks to each other, but to gather consistent performance from participants, they completed all three tasks in the same order, beginning with the D2 task, followed by the SWM task, and finishing with the DMTS task. As the attention of participants is likely to wane over time, it was important that all participants complete the tasks in the same order. In both studies, we recruited 100 participants per condition. Previous work on predicting PHQ-9 scores using smartphone sensors used *n* = 28 (Canzian and Musolesi, 2015), *n* = 79 (Farhan et al., 2016), *n* = 83 (Wang et al., 2018), *n* = 126 (Wahle et al., 2016), and *n* = 138 (Chikersal et al., 2021). We used the heuristic of 100 people per condition based on the sample sizes in this previous literature (Lakens, 2021). In Study One, we recruited *n* = 300, but there were missing data logs for 3 people, leaving *n* = 297 with complete data. In Study Two, we recruited *n* = 100, but there were missing data logs for 8 people, leaving *n* = 92 with complete data.

### 2.4. Data Filtering

Because data were gathered online in uncontrolled contexts, we needed to remove spurious responses from participants who did not engage with the experiment (e.g., were clicking randomly) and from potential bots. We followed best practices for collecting and cleaning online data (Meade and Craig, 2012; Buchanan and Scofield, 2018). In both studies, we filtered out participants who completed the study too quickly, defined as less than 1 s per item on more than two scales, which indicated a lack of attention in completing responses. Second, we removed participants who violated a zero variance filter, indicating there was zero diversity in their responses (they simply repeated the same response), on more than two scales. Third, we ran a variance filter to detect responses from participants that were more than three standard deviations above the mean variance, indicating that they were clicking randomly, on more than two scales.

In Study One, the filtering process removed 27 participants, leaving 269 valid responses (D2 = 90; DMTS = 92, SWM = 87) that were processed and used for further analyses. Participants were (female = 109, male = 160) aged 18 to 72 (*M* = 36.665; *Mdn* = 34.000; *SD* = 11.377). In Study Two, we removed 2 participants, leaving 90 valid participants used for further analysis. Participants (female = 33, male = 57) were aged 25 to 68 (*M* = 37.944; *Mdn* = 35.500; *SD* = 11.155).

### 2.5. Statistical Analyses

We conducted multiple regression analyses using the measures from the digital tool to predict PHQ-9 scores, with a significance threshold of α = 0.05. Using hierarchical regressions, we controlled for age and gender by entering them in the first block and adding the measures of interest in the second block. We calculated separate regression models for each of the measures of the three tasks to assess suitability of the measures for prediction while accounting for their similarity resulting in substantial shared variance, and also a combined model in Study Two, in which the predictions are made by the non-shared rather than the shared variance within the set of predictors. We report unstandardized regression coefficients (*B*) with standard errors (*se B*), standardized regression coefficients (β), *t*-values, and *p*-values for individual predictors and *R*^2^-values, *F, p*-values, *R*^2^ change (Δ*R*^2^), and F change (ΔF) for the regression models to demonstrate goodness of fit. We tested for multicollinearity using variance inflation factors (VIF), which were substantially lower than values that have been suggested as thresholds for necessary corrections (Kock and Lynn, 2012) (Study One: all VIFs < 1.119, Study Two, Tasks in Isolation: all VIFs < 1.146, Study Two, Tasks in Combination: all VIFs < 1.504). JASP 0.14.1 was used for data analysis (JASP Team, 2020).

## 3. Results

### 3.1. Study One: Tasks in Isolation

In Study One, we investigated the tasks in isolation (between-subjects design) and the suitability of their measures to predict PHQ-9 scores. Table 1 shows the descriptive statistics.

**Table 1:** Descriptive statistics for Study One and Study Two.

	**N_1_**	**Mean_**1**_**	**Std.Dev._**1**_**	**N_2_**	**Mean_2_**	**Std.Dev._2_**
PHQ-9	269	6.836	6.913	90	7.022	6.892
Number of items	90	426.789	115.491	90	419.233	94.922
Number of correctly marked stimuli	90	172.344	48.566	90	168.133	43.076
Number of omission errors	90	21.222	27.834	90	22.211	22.142
Number of commission errors	90	20.811	49.508	90	20.600	37.195
Total number of errors	90	42.033	57.181	90	42.811	52.897
Error rate	90	9.654	11.448	90	10.244	11.217
Number of correct choices	92	32.533	6.046	90	31.700	6.523
Average latency	92	3241.195	1330.385	90	4128.393	6994.663
Number of color errors	92	1.902	2.589	90	1.933	2.508
Number of shape errors	92	4.261	2.897	90	4.767	3.006
Number of unrelated errors	92	1.304	1.880	90	1.600	2.350
Number of between errors	87	84.563	46.407	90	92.278	55.287
Number of within errors	87	16.414	39.549	90	19.822	38.204
Strategy score	87	40.046	8.158	90	40.511	8.260

#### 3.1.1. D2 Test of Attention

Table 2 shows results for the D2 task. First, PHQ-9 scores had a significant negative association with age, while gender was not a significant predictor. Then, controlling for age and gender, PHQ-9 scores were not significantly predicted by the *number of items, number of correctly marked stimuli*, or the *number of omission errors*. In contrast, there were significant effects for the other measures. PHQ-9-scores were indicated by a higher *number of commission errors, total number of errors*, and *error rate*.

**Table 2:** Isolated D2 regression results.

**Model**		***B***	***se B***	**β**	***t***	***p***	**R^2^**	***F***	***p* model**	**ΔR^2^**	**ΔF**
H_0_	(Intercept)	14.505	2.585		5.612	<0.001					
	Age	−0.217	0.069	−0.329	−3.137	0.002					
	Gender	−0.051	0.696	−0.008	−0.073	0.942	0.107	5.212	0.007	0.107	5.212
H_1_	Number of items	5.439e−4	0.006	0.009	0.089	0.929	0.107	3.438	0.020	0.000	0.008
H_1_	Number of correctly marked stimuli	−8.436e−4	0.014	−0.006	−0.059	0.953	0.107	3.436	0.020	0.000	0.004
H_1_	Number of omission errors	0.011	0.025	0.046	0.443	0.659	0.109	3.508	0.019	0.002	0.196
H_1_	Number of commission errors	0.037	0.013	0.278	2.808	0.006	0.182	6.377	<0.001	0.075	7.883
H_1_	Total number of errors	0.030	0.012	0.260	2.626	0.010	0.173	6.010	<0.001	0.066	6.898
H_1_	Error rate	0.184	0.057	0.316	3.242	0.002	0.204	7.358	<0.001	0.097	10.512

*H_0_ is the results for age and gender; H_1_ shows the results of adding the measures individually into the second block*.

#### 3.1.2. Delayed Matching to Sample

Table 3 shows results for the DMTS task. Age and gender were non-significant. Controlling for these variables, all measures were significant predictors for PHQ-9 scores. Higher PHQ-9 scores were negatively associated with the *number of correct choices* and accordingly positively associated with *number of color errors, number of shape errors*, and *number of color+shape errors*. Further, *average latency* predicted PHQ-9 scores.

**Table 3:** Isolated DMTS regression results.

**Model**		***B***	***se B***	**β**	***t***	***p***	**R^2^**	***F***	***p* model**	**ΔR^2^**	**ΔF**
H_0_	(Intercept)	4.876	2.607		1.870	0.065					
	Age	0.050	0.067	0.078	0.747	0.457					
	Gender	1.037	0.764	0.142	1.357	0.178	0.025	1.143	0.323	0.025	1.143
H_1_	Number of correct choices	−0.483	0.113	−0.413	−4.253	<0.001	0.191	6.937	<0.001	0.166	18.084
H_1_	Average latency	0.001	5.539e−4	0.280	2.683	0.009	0.099	3.215	0.027	0.074	7.199
H_1_	Number of color errors	0.938	0.270	0.343	3.468	<0.001	0.142	4.865	0.004	0.117	12.024
H_1_	Number of shape errors	0.694	0.254	0.284	2.735	0.008	0.101	3.311	0.024	0.076	7.480
H_1_	Number of color+shape errors	1.573	0.362	0.418	4.341	<0.001	0.197	7.197	<0.001	0.172	18.845

*H_0_ is the results for age and gender; H_1_ shows the results of adding the measures individually into the second block*.

#### 3.1.3. Spatial Working Memory

Table 4 shows results for the SWM task. For these participants, age and gender were significant predictors. Age had a negative association with PHQ-9 scores and was higher for female participants (*M* = 8.656) than for male participants (*M* = 5.727). Controlling for age and gender, *strategy score* was a positive, significant predictor for PHQ-9 scores while effects for *number of between errors* and *number of within errors* did not reach significance.

**Table 4:** Isolated SWM regression results.

**Model**		***B***	***se B***	**β**	***t***	***p***	**R^2^**	***F***	***p* model**	**ΔR^2^**	**ΔF**
H_0_	(Intercept)	13.607	2.204		6.174	<0.001					
	Age	−0.172	0.056	−0.314	−3.089	0.003					
	Gender	−1.682	0.741	−0.231	−2.270	0.026	0.138	6.742	0.002	0.138	6.742
H_1_	Number of between errors	0.027	0.015	0.174	1.727	0.088	0.168	5.594	0.002	0.030	2.982
H_1_	Number of within errors	0.011	0.018	0.060	0.586	0.559	0.142	4.574	0.005	0.004	0.344
H_1_	Strategy score	0.174	0.086	0.201	2.016	0.047	0.179	6.013	<0.001	0.040	4.064

*H_0_ is the results for age and gender; H_1_ shows the results of adding the measures individually into the second block*.

### 3.2. Study Two: Tasks in Isolation

In Study Two, participants completed all three tasks. First, we investigated measures in isolation to confirm the suitability of individual metrics and tasks to predict PHQ-9 scores. As the same set of participants engaged in all tasks, the null model including age and gender was the same for all tasks. For this sample, PHQ-9 scores were not significantly predicted by gender, but showed a significant, negative association with age.

#### 3.2.1. D2 Test of Attention

Controlling for age and gender, regression models for the D2 task measures showed mostly consistent results to Study One. Again, PHQ-9 scores were not significantly predicted by the *number of items* or *number of correctly marked stimuli* but significantly associated with higher *number of commission errors, total number of errors*, and *error rate*. Further, and in contrast to Study One, the relationship between PHQ-9 scores and the *number of omission errors* was also significant and positive in this study. Table 5 shows these results.

**Table 5:** Combined D2 regression results.

**Model**		***B***	***se B***	**β**	***t***	***p***	**R^2^**	***F***	***p* model**	**ΔR^2^**	**ΔF**
H_0_	(Intercept)	13.966	2.571		5.433	<0.001					
	Age	−0.180	0.064	−0.291	−2.799	0.006					
	Gender	−0.487	0.739	−0.068	−0.659	0.512	0.083	3.944	0.023	0.083	3.944
H_1_	Number of items	−0.002	0.008	−0.030	−0.292	0.771	0.084	2.630	0.055	0.001	0.085
H_1_	Number of correctly marked stimuli	−0.031	0.016	−0.195	−1.931	0.057	0.121	3.955	0.011	0.038	3.728
H_1_	Number of omission errors	0.103	0.030	0.329	3.380	0.001	0.191	6.752	<0.001	0.108	11.424
H_1_	Number of commission errors	0.072	0.019	0.388	3.831	<0.001	0.217	7.934	<0.001	0.134	14.673
H_1_	Total number of errors	0.053	0.013	0.409	4.183	<0.001	0.238	8.962	<0.001	0.155	17.501
H_1_	Error rate	0.279	0.059	0.454	4.692	<0.001	0.270	10.604	<0.001	0.187	22.017

*H_0_ is the results for age and gender; H_1_ shows the results of adding the measures individually into the second block*.

#### 3.2.2. Delayed Matching to Sample

The results for the regression models for the DMTS task (see Table 6) were mostly consistent with those from Study One. Again, PHQ-9 scores were negatively and significantly associated with *number of correct choices* and accordingly predicted by *number of color errors, number of shape errors*, and *number of color+shape errors* with significant and positive relationships. In this study, the relationship of *average latency* with PHQ-9 scores did not reach significance.

**Table 6:** Combined DMTS regression results.

**Model**		***B***	***se B***	**β**	***t***	***p***	**R^2^**	***F***	***p* model**	**ΔR^2^**	**ΔF**
H_0_	(Intercept)	13.966	2.571		5.433	<0.001					
	Age	−0.180	0.064	−0.291	−2.799	0.006					
	Gender	−0.487	0.739	−0.068	−0.659	0.512	0.083	3.944	0.023	0.083	3.944
H_1_	Number of correct choices	−0.458	0.102	−0.433	−4.489	<0.001	0.257	9.926	<0.001	0.174	20.153
H_1_	Average latency	1.589e−4	1.020e−4	0.161	1.557	0.123	0.108	3.481	0.019	0.025	2.424
H_1_	Number of color errors	1.212	0.260	0.441	4.656	<0.001	0.268	10.481	<0.001	0.185	21.678
H_1_	Number of shape errors	0.490	0.234	0.214	2.094	0.039	0.128	4.194	0.008	0.044	4.387
H_1_	Number of color+shape errors	1.316	0.287	0.449	4.590	<0.001	0.264	10.260	<0.001	0.180	21.070

*H_0_ is the results for age and gender; H_1_ shows the results of adding the measures individually into the second block*.

#### 3.2.3. Spatial Working Memory

Table 7 shows results for the SWM task. PHQ-9 scores had positive, significant relationships with all measures: *number of between errors, number of within errors*, and *strategy score*.

**Table 7:** Combined SWM regression results.

**Model**		***B***	***se B***	**β**	***t***	***p***	**R^2^**	***F***	***p* model**	**ΔR^2^**	**ΔF**
H_0_	(Intercept)	13.966	2.571		5.433	<0.001					
	Age	−0.180	0.064	−0.291	−2.799	0.006					
	Gender	−0.487	0.739	−0.068	−0.659	0.512	0.083	3.944	0.023	0.083	3.944
H_1_	Number of between errors	0.040	0.012	0.319	3.246	0.002	0.183	6.429	<0.001	0.100	10.534
H_1_	Number of within errors	0.050	0.018	0.276	2.733	0.008	0.156	5.315	0.002	0.073	7.471
H_1_	Strategy score	0.223	0.083	0.267	2.692	0.009	0.154	5.233	0.002	0.071	7.245

*H_0_ is the results for age and gender; H_1_ shows the results of adding the measures individually into the second block*.

### 3.3. Study Two: Tasks in Combination

The previous analyses focused on validating the measures' suitability for predicting PHQ-9 scores individually. To complement this, we evaluated whether a digital assessment tool consisting of multiple tests might be even more powerful, i.e., better at predicting PHQ-9 scores.

For that purpose, we selected one metric from each task and combined them in a multiple regression. To identify the metrics that were most discriminating and individually useful, we conducted a principal component analysis (oblimin rotation) with three factors on the measures that were significant predictors in the linear regressions [$\chi_{\left(25\right)}^{2}=3325.12,p<0.001$]. As Table 8 shows, the measures loaded on factors associated with their task (i.e., D2, DMTS, SWM); from these, we selected the metric for each task that loaded highest on the factor associated with a task metric for use in the multiple regression: *total number of errors* (D2), *number of correct choices* (DMTS), and *number of within errors* (SWM).

**Table 8:** Component loadings.

	**RC1**	**RC2**	**RC3**	**Uniqueness**
Number of omission errors	0.862			0.313
Number of commission errors	0.916			0.145
Total number of errors	1.005			0.008
Error rate	0.949			0.058
Number of correct choices		−0.941		0.002
Number of color errors		0.672		0.284
Number of shape errors		0.928		0.287
Number of color+shape errors		0.709		0.171
Number of between errors			0.764	0.210
Number of within errors			0.811	0.393
Total strategy score			0.769	0.440

*Factors related to D2 loaded on RC1, DMTS on RC2, and SWM on RC3*.

Then, we conducted a hierarchical multiple regression analysis, again controlling for age and gender at the null model, and then entering the three predictors at the first level (forced entry). Table 9 shows the results for this model. The results show that all three measures were significant predictors for PHQ-9 scores, indicating their individual value in a combined model. This model accounted for 34.4% of the variance in PHQ-9 scores, substantially outperforming all models with individual predictors and highlighting the value of the digital assessment toolbox with all three tasks.

**Table 9:** Regression results for all tasks combined.

**Model**		***B***	***se B***	**β**	***t***	***p***	**R^2^**	***F***	***p* model**	**ΔR^2^**	**ΔF**
H_0_	(Intercept)	13.966	2.571		5.433	<0.001					
	Age	−0.180	0.064	−0.291	−2.799	0.006					
	Gender	−0.487	0.739	−0.068	−0.659	0.512	0.083	3.944	0.023	0.083	3.944
H_1_	(Intercept)	16.176	4.516		3.582	<0.001					
	Age	−0.069	0.058	−0.112	−1.180	0.241					
	Gender	−0.649	0.637	−0.091	−1.019	0.311					
	Total number of errors (D2)	0.035	0.014	0.266	2.493	0.015					
	Number of correct choices (DMTS)	−0.272	0.115	−0.257	−2.371	0.020					
	Number of within errors (SWM)	0.038	0.017	0.213	2.316	0.023	0.344	8.799	<0.001	0.261	11.119

*H_0_ is the results for age and gender; H_1_ shows the results of adding the measures simultaneously into the second block*.

## 4. Discussion

### 4.1. Summary of Findings

Through two experiments, we consistently and significantly predicted PHQ-9 scores from error measures of attention tasks gathered online and *in situ*. In almost all models (except for isolated DMTS), age showed an overall negative association with PHQ-9 scores, consistent with prior knowledge on depression over the lifespan (Patten et al., 2006; Tomitaka et al., 2018). Our results conforming to expectations does lend support for the accurate self-report of depression using the PHQ-9 in our sample.

From the attention tests themselves, there were several good predictors of PHQ-9. For the D2 task, the *Number of commission errors, total number of errors*, and *error rate* were positive, significant predictors of self-reported depression in both studies. For the DMTS task, PHQ-9 scores were predicted by *number of correct choices* (negative relationship) and by *number of color errors, number of shape errors*, and *number of color+shape errors* (positive relationship) in both studies. For the SWM task, *strategy score* had a positive, significant relationship with PHQ-9 scores in both studies. Further, in the first study, we saw significant predictions from *average latency* in the DMTS, and in the second study, we additionally saw significant predictions from the *number of omission errors* in the D2 and the *number of between errors* and *number of within errors* in the SWM task.

While the between and within errors for SWM did not strongly predict PHQ-9 scores in Study One, they did so in Study Two. Table 1 suggests that participants made more errors due to decreased attention in Study Two, when the SWM happened after the D2, which might suggest that these measures are good indicators only in some instances, e.g., when participants have decreased attention or are already fatigued. However, this idea requires further investigation. Similarly, the DMTS was performed last in Study Two, and the significant results for average latency seen in Study One did not replicate. However, Table 1 shows that the average latency was slightly elevated in Study Two, in which the DMTS was done last, but also that the standard deviation was much higher, suggesting greater variance in latency responses.

Although there have been previously demonstrated relationships between both error metrics and timing metrics with depression, our findings point more to robustness in error-related measures in our experiment. We suspected in advance that this might be the case, and we posit that there are fewer repercussions of the uncontrolled environment in error measures than in response times, which can be affected by differences in hardware (e.g., known differences between mice and trackpads; Soukoreff and MacKenzie, 2004), software (e.g., cursor acceleration settings; Casiez et al., 2008), and networks (e.g., network latencies; Long and Gutwin, 2018). Although our results did not demonstrate strong relationships between timing variables and depression, we believe that our findings do not lie in contrast with earlier work on cognitive deficits in depression. We require more work to test the relationship between timing and depression to make claims on a theoretical level. It is possible that individuals interacting with digital assessment tools in their home context and on their variable computing systems just behave in a particular way, in which timing is less indicative of depression than error-based measures. Interestingly, speed-accuracy tradeoffs mean that participants often prioritize one of speed or accuracy, and recent work suggests that for attention tasks in particular, measures of accuracy (i.e., errors) are not consistently associated with measures of response time (Hedge et al., 2018a).

In Study Two, the combined model (with one metric from each task) outperformed all the individual models, and accounted for 34.4% of the variance in PHQ-9 scores, indicating that the combination of metrics has value over simply looking at metrics in isolation. Although these error metrics from the different tasks are related, the non-shared variance in the multiple regression model generated a better prediction than any of the isolated models. Further, the attention metrics explained a greater proportion of variance than age and gender alone, which in a single model explained only 8.3% of the variance in PHQ-9 scores (see Table 9). The addition of the scores from the digital tool were necessary to explain over a third of the variance in PHQ-9 scores.

### 4.2. Contextualization and Implications of Findings

The assessment of attention is a challenging undertaking, but is important as attention is a cognitive function that is indicative of human development and relates to mental health. Beyond depression, attention and attentional control are both related to a variety of other cognitive deficits, such as attention-deficit/hyperactivity disorder (Barkley, 1997) and dementia (Perry and Hodges, 1999), and also to human capabilities, such as reading ability (Franceschini et al., 2012). As accurately measuring attention could help assess and diagnose a number of common disorders, the success of our digital tool has implications beyond our intended goal of assessing depression remotely. Classification systems like the DSM-V (American Psychiatric Association, 2013) standardize diagnoses of mental health disorders; however, comorbidity of mental health symptoms is not the exception, but the norm (Kessler et al., 2005). Relevant to our work, there is high comorbidity between symptoms of depression and anxiety (Kircanski and Gotlib, 2015) and our results do not attempt to differentiate between these conditions. More work is needed to move toward transdiagnostic approaches to assessment that transcend categorical classification, but rather focus on underlying process mechanisms to inform diagnosis (Frank and Davidson, 2014). Future work can consider whether behavioral biomarkers can contribute to disentangling symptoms of multiple comorbid disorders.

It is challenging to compare our findings to prior work; there are no previous approaches that also used regression to predict PHQ-9 scores from performance data on a suite of tasks. Two meta-analyses on attentional deficits and depression report effect sizes (Cohen's d) that reflect the difference between performance on attention tasks between people with depression and healthy controls. These effect sizes range from 0.34 to 0.65 (Rock et al., 2014) and 0.59 (Wang et al., 2020), which indicate significant moderate effects. We cannot directly compare, as we do not examine group differences, but rather predict a range of PHQ-9 scores from a set of error scores. However, in calculating the effect size *f*^2^ of the addition of the three error measures in our multiple regression from Study Two, we have an *f*^2^ = 35, which indicates a large effect. It is not surprising that our effect size is large, as we used three measures in combination. The effect sizes for the individual predictors (which are a better comparator to the results from the meta-analyses) are slightly smaller, but still indicate moderate to large effects.

Of significance is that our approach was to use participants' own computers in the uncontrolled environment of their home. Measuring attention can be challenging in the lab; however, doing so in the uncontrolled context of people's own homes is even more difficult. As previously argued, the differences in hardware affect display latencies, screen resolutions, and visual angle, whereas the differences in software affect interactive input. By focusing on error-related measures, and not on response latencies or reaction times, we minimized the effects of variations in computing systems. However, there remain differences in the context of participants' homes that were uncontrolled; interruptions such as pets, children, auditory interruptions, and multi-tasking are all not controlled in our experiment and likely influenced the results.

Our approach uses continuous prediction, rather than binary classification. This regression approach means that we are not classifying people into PHQ-9 levels, but are predicting their score along a range. Classification is possible, but would necessitate machine learning techniques, such as those used in the passive sensing approaches of smartphone data (Chikersal et al., 2021) or social media data (De Choudhury et al., 2013). Although there is benefit in classification, a first step is to demonstrate a consistent statistical relationship between the metrics and PHQ-9 scores, which we provide in this paper.

Another difference between our approach and the passive sensing approaches described earlier is that our tool uses an explicit method of gathering data. The work on detecting depression from smartphones or social media assumes that people are using their phones and social media for other purposes, but then harnesses these signals for use as a depression detector. This passive sensing approach has the advantage of being applicable to any user of a smartphone or social media, which would reach the majority of the population. Our active sensing approach requires that people engage explicitly with our digital tool, and thus has a much smaller reach. However, by requiring explicit use, our tool also brings explicit consent of participation to the fore. Profiling technologies, such as those that detect personality disorders or mental health problems from stealthy methods such as eyetracking (e.g., Berkovsky et al., 2019) or social media use (e.g., Reece and Danforth, 2017) have been criticized for realizing a dystopian future in which marginalized populations that are already stigmatized experience further discrimination and harm from artificial intelligence and algorithmic decision making (Alkhatib, 2021). How data derived from digital sources is gathered, and for what purpose, is part of a larger discussion on the ethics of data use, dark patterns of interaction, and tech ethics (Kitchin, 2014; Mittelstadt et al., 2016). Although consent is not built into our digital tool, the explicit approach to gathering data does reduce the potential for large-scale unethical misuse.

### 4.3. Limitations and Future Work

Although our experiment suggests that remote assessment of depression has potential, there are several limitations to our study.

First, we assess depression using self-reported PHQ-9 scores. Although this is the gold standard self-report tool for clinical assessment (Kroenke et al., 2001), there are limitations with self-report. Answers can be affected by social desirability biases (Lavrakas, 2008b), can show unintended variance as has been demonstrated from test-retest reliability (Lavrakas, 2008c)) and respondents can be fatigued from answering many items on several questionnaires (Lavrakas, 2008a). Further, we predict the PHQ-9 score, and not the PHQ-9 level. To be effectively used as a screening tool, future work should determine if the biomarkers can be used to predict PHQ-9 level, using machine learning classification approaches, essentially indicating the severity of the symptoms. In future work, our digital assessment tool should be extended into clinical samples to predict diagnoses of depression as compared to a control group.

Second, our data was collected online, by intention. As our goal was to develop tools that can aid in remote assessment that will be undertaken *in situ*, testing our tool's validity in an uncontrolled environment was a necessary methodological approach. However, online studies can be subject to variations in response quality, and our tool should also be assessed in a controlled laboratory context.

Third, our tool was able to explain 34.4% of variance in a multiple regression model. Although this is, in practice, a large amount—over a third of the variance in PHQ-9 scores were explained by solely age, gender, and three attention metrics—additional measures may need to be incorporated for our tool to be used as a classification tool.

Fourth, some of the relationships between predictors and depression scores did not hold as expected (e.g., *number of items* in D2) or were inconsistent (e.g., *number of within errors* as non-significant in Study One but significant in Study Two). At this stage, we can only speculate about the reasons. For instance, it may be that longer exposure and more tasks are necessary for individuals to perform enough errors in SWM that they are indicative of depression scores. Thus, measures may be significant in Study Two, where participants completed the SWM after the D2 task. Alternatively, inconsistent or null effects may be due to specifics of our implementation and thus require further investigation. While our work does not aim to or allow for interpretation on a theoretical level, it is important to conduct further work to investigate inconsistent and null effects.

## 5. Conclusions

In this paper, we describe the design and evaluation of a non-clinical digital assessment tool that integrates digital biomarkers of depression. Based on three standard cognitive tasks (D2 Test of Attention, Delayed Matching to Sample Task, Spatial Working Memory Task) on which people with depression have been known to perform differently than a control group, we iteratively designed a digital assessment tool that could be deployed outside of laboratory contexts, in uncontrolled home environments on computer systems with widely varying system characteristics (e.g., displays resolution, input devices). We conducted two online studies, in which participants used the assessment tool in their own homes, and completed subjective questionnaires including the Patient Health Questionnaire (PHQ-9)—a standard self-report tool for assessing depression in clinical contexts. In a first study (*n* = 269), we demonstrate that each task can be used in isolation to significantly predict PHQ-9 scores. In a second study (*n* = 90), we replicate these results and further demonstrate that when used in combination, behavioral metrics significantly predicted PHQ-9 scores, even when taking into account demographic factors known to influence depression such as age and gender. A multiple regression model explained 34.4% of variance in PHQ-9 scores with several behavioral metrics from the tool providing unique and significant contributions to the prediction.

Our findings can help inform clinician assessment of depression with objective digital biomarkers of depression that are gathered easily on home computers outside of the clinical context. We contribute to the design of digital biomarkers of depression, which can be used in concert with existing assessments to promote accessible, equitable, early, ongoing, and large-scale assessment of depression.

## Data Availability Statement

The raw data supporting the conclusions of this article will be made available by the authors, without undue reservation.

## Ethics Statement

The studies involving human participants were reviewed and approved by Behavioural Research Ethics Committee at the University of Saskatchewan (BEH 17-418). The patients/participants provided their written informed consent to participate in this study.

## Author Contributions

RM led the research, designed the tool and experiment, conducted the analysis, and wrote the manuscript. MB contributed to the idea, the design of the tool, the experiment design, and the analysis plan. SV implemented the tool, designed the interfaces, and gathered the data for both studies. KW contributed to the assessment of attention online and wrote parts of the manuscript. ER implemented the prototype of the DMTS task and generated the visual stimuli. PB implemented the prototype of the SWM task. JF contributed to the experiment design, conducted the data analysis, and wrote the manuscript. All authors edited the manuscript.

## Funding

Funding was provided by the Natural Sciences and Engineering Research Council of Canada through the Discovery Grant program and the E.W.R. Steacie Memorial Fellowship program. This publication was partially supported by the VENI research project VI.Veni.202.171, financed by the Dutch Research Council (NWO).

## Conflict of Interest

The authors declare that the research was conducted in the absence of any commercial or financial relationships that could be construed as a potential conflict of interest.

## Publisher's Note

All claims expressed in this article are solely those of the authors and do not necessarily represent those of their affiliated organizations, or those of the publisher, the editors and the reviewers. Any product that may be evaluated in this article, or claim that may be made by its manufacturer, is not guaranteed or endorsed by the publisher.
